# The Role of the Anti-Inflammatory Cytokine Interleukin-10 in Tissue Fibrosis

**DOI:** 10.1089/wound.2019.1032

**Published:** 2020-02-07

**Authors:** Emily H. Steen, Xinyi Wang, Swathi Balaji, Manish J. Butte, Paul L. Bollyky, Sundeep G. Keswani

**Affiliations:** ^1^ Department of Surgery, Baylor College of Medicine, Houston, Texas.; ^2^ Laboratory for Regenerative Tissue Repair, Texas Children's Hospital, Houston, Texas.; ^3^ Division of Immunology, Allergy, and Rheumatology, Department of Pediatrics, University of California, Los Angeles, Los Angeles, California.; ^4^ Division of Infectious Diseases, Department of Medicine, Stanford University School of Medicine, Stanford, California.; ^5^ Division of Pediatric Surgery, Department of Surgery, Texas Children's Hospital, Houston, Texas.

**Keywords:** interleukin-10, hyaluronan, extracellular matrix, fibrosis, cell biology

## Abstract

**Significance:** Fibrosis is the endpoint of chronic disease in multiple organs, including the skin, heart, lungs, intestine, liver, and kidneys. Pathologic accumulation of fibrotic tissue results in a loss of structural integrity and function, with resultant increases in morbidity and mortality. Understanding the pathways governing fibrosis and identifying therapeutic targets within those pathways is necessary to develop novel antifibrotic therapies for fibrotic disease.

**Recent Advances:** Given the connection between inflammation and fibrogenesis, Interleukin-10 (IL-10) has been a focus of potential antifibrotic therapies because of its well-known role as an anti-inflammatory mediator. Despite the apparent dissimilarity of diseases associated with fibrotic progression, pathways involving IL-10 appear to be a conserved molecular theme. More recently, many groups have worked to develop novel delivery tools for recombinant IL-10, such as hydrogels, and cell-based therapies, such as *ex vivo* activated macrophages, to directly or indirectly modulate IL-10 signaling.

**Critical Issues:** Some efforts in this area, however, have been stymied by IL-10's pleiotropic and sometimes conflicting effects. A deeper, contextual understanding of IL-10 signaling and its interaction with effector cells, particularly immune cells, will be critical to future studies in the field.

**Future Directions:** IL-10 is clearly a gatekeeper of fibrotic/antifibrotic signaling. The development of novel therapeutics and cell-based therapies that capitalize on targets within the IL-10 signaling pathway could have far-reaching implications for patients suffering from the consequences of organ fibrosis.

**Figure:**
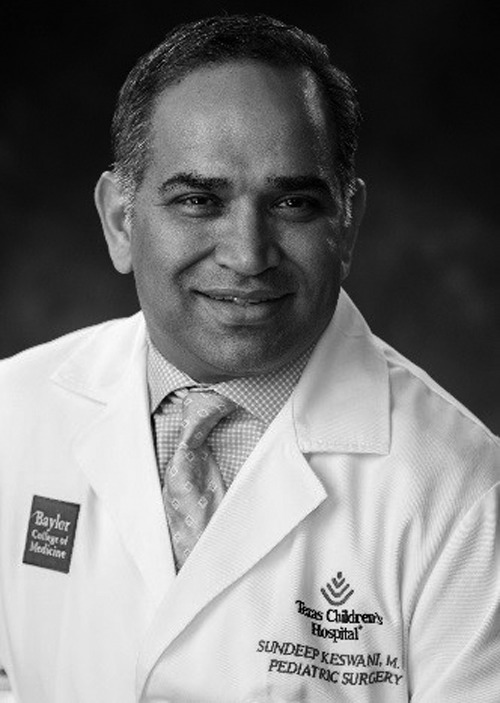
**Sundeep G. Keswani, MD, FACS, FAAP**

## Scope and Significance

Fibrosis results in a loss of structural integrity and function and is the endpoint of many chronic diseases in multiple organs. Interleukin-10 (IL-10) is known as an anti-inflammatory cytokine, but it also appears to function as a conserved gatekeeper of fibrotic processes. In this study, we first discuss the pathogenesis of fibrosis and IL-10 signaling mechanisms. We then discuss literature on fibrosis and IL-10 in various tissues and novel therapies that use IL-10 signaling as an antifibrotic strategy.

## Translational Relevance

Although prior work on IL-10 in fibrosis comes from many fields in studying many different disease processes, there appear to be unifying themes within the literature. This review seeks to consolidate the literature on IL-10 signaling and multiorgan fibrosis with the intention of identifying common pathways between various pathologies, which may lead to novel therapeutic targets.

## Clinical Relevance

As the final common pathway of many disease states, fibrosis has relevance to clinicians from all specialties and disciplines, as well as to the many patients they treat. An improved understanding of IL-10 as a fibrogenic regulator, leading to new therapeutic targets, could vastly improve outcomes and quality of life for patients with diseases marked by dysregulated or excessive extracellular matrix (ECM) deposition. Furthermore, new research on the cellular context of IL-10 signaling has led to novel cell-based therapies that have the potential to revolutionize the treatment of fibrosis in multiple organs.

## Background and Overview

### Cellular injury and fibrogenesis

**Figure 1.**
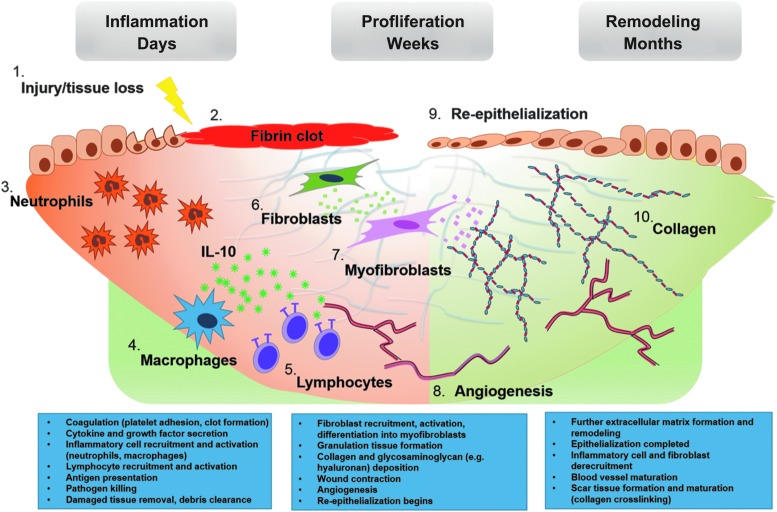
Wound healing and fibrogenesis. Wound healing progresses in a well-defined series of steps. After tissue injury (1) coagulation begins, ultimately resulting in the formation of a fibrin clot (2). Damaged tissue or detected pathogens spur the release of local cytokine and growth factor release, beginning the inflammatory phase of wound healing. These “danger signals” result in the recruitment of local and circulating innate immune cells, neutrophils (3) and macrophages (4) being predominant initially. These phagocytes begin antigen presentation and thereby recruit and activate effector cells of the adaptive immune system (5). IL-10 is broadly expressed by immune cells, but the predominant cellular sources are macrophages and T cell subsets (*i.e*., T helper 2 and regulatory T cells).^101,102^ Pathogen and damaged tissue clearance is the ultimate result of the inflammatory phase, staging the wound bed for regrowth. The proliferative phase is characterized by the activities of the fibroblast (6), which secretes the ECM components that provide the scaffolding for regenerated tissue. Granulation tissue, composed of immature blood vessels (8) and loose connective tissue fibers, begins to fill the wound, providing a structure within which fibroblasts can act and upon which epithelial cells migrate (9). Fibroblasts are induced by various cytokines and growth factors to differentiate into myofibroblasts (7), strengthening the wound by depositing collagen fibers, glycosaminoglycans, and other structural macromolecules. The myofibroblast phenotype is also contractile, acting to hasten wound closure. At this point, the remodeling phase begins, as newly created structures undergo maturation and strengthening or are pruned away. This stage can last from months to years. The initially deposited collagen III is replaced by collagen I, and collagen bundling and crosslinking (10) serves to further increase the tensile strength of the wound, while also resulting in what we recognize as scar tissue formation. Inflammatory cells and fibroblasts are no longer recruited, and many of those present in the wound bed undergo apoptosis. This gradual quiescence concludes the wound healing process and prevents the continued production of scar tissue, which could lead to tissue dysfunction. ECM, extracellular matrix; IL-10, interleukin-10.

Despite the diverse means by which the body sustains injury, most organs repair themselves through a common pathway that ultimately leads to the formation of collagen deposition or fibrogenesis (Fig. 1). Therefore, it may be reasonable to expect that there are conserved cellular and molecular mechanisms that govern fibrosis among several organ systems. The recurrent players and cellular pathways involved give rise to the overarching and interconnected themes behind the complex process of restoring homeostasis.

The tissue response to injury is initiated after primary damage to the organ, beginning with the activation of the coagulation pathway, a rapid phase aimed at limiting blood loss. This is then followed by acute inflammation with activation of effector cells, specifically innate immune cells such as neutrophils and macrophages that release damage-associated signals and begin antigen presentation. This then initiates an adaptive immune response, which more directly addresses the insult and promotes ECM production and angiogenesis. The newly deposited matrix serves to temporarily restore structural integrity to the tissue and, over a period of days or weeks, is then remodeled and ultimately results in scar formation. The postinjury inflammatory cascade is a critical step in normal wound healing and fibrosis; tissue repair involving attenuated, exaggerated, or prolonged immune responses is known to result in altered fibrotic phenotypes. Therefore, a better understanding of the potential common pathways in the inflammatory process may yield therapies aimed at modulating that process, with significant benefit to patients.^1,2^ There is a substantial body of data that implicates the cytokine IL-10 in the regulation of tissue inflammatory responses and, more recently, in governing fibrogenesis in several organ systems.^3^

### Interleukin-10, inflammation, and extracellular matrix remodeling

Interleukin-10 (IL-10) is a pleomorphic cytokine with diverse phenotypic effects. Initially discovered as a product of T helper 2 cells that inhibited T helper 1 cell activation, it is now known to be produced by almost all species of activated immune cells, including B cells, mast cells, granulocytes (*e.g*., neutrophils, basophils, eosinophils), macrophages, dendritic cells, and multiple T cell subsets.^4^ Its principal actions are primarily considered anti-inflammatory, inhibitory, or self-regulating, in that IL-10 appears to be a potent negative feedback regulator that effects the control and resolution of inflammation via autocrine and paracrine mechanisms (Table 1). This immunosuppressive effect is broad and occurs at both the cellular and humoral levels, although there are two dominant means by which IL-10 limits potentially damaging inflammatory responses: (1) inhibiting antigen presentation by dendritic cells and (2) inhibiting macrophage activation and infiltration into the site of injury, with the secondary effect of attenuating proinflammatory cytokine expression.^4^ At the cellular level, IL-10 is believed to act as a posttranscriptional regulatory agent to suppress the messenger RNA (mRNA) stabilizing protein HuR (human antigen R), promoting the specific destabilization of inflammatory cytokine mRNA.^5^ In addition, IL-10 is thought to inhibit apoptotic signaling pathways, such as the p38 MAPK (mitogen-activated protein kinase) pathway, via signal transducer and activator of transcription 3 (STAT3)-dependent signaling, thereby limiting tissue death and organ dysfunction after injury.^6–8^

**Table 1.** The Multifunctionality of Interleukin-10 in Organ Fibrosis

Organ	Fibrotic Disease Processes	Role of IL-10	References/Reviews
Skin	Normal scarring, hypertrophic scarring, SSc, psoriasis	Beneficial	^16,21–23,103,104^
	Normal scarring, hypertrophic scarring, SSc	Maladaptive	^105,106^
Heart	Systolic and diastolic HF, cardiac fibrosis (post-MI, isoproterenol-induced), autoimmune myocarditis	Beneficial	^3,7,30,33,36,107^
	Diastolic HF	Maladaptive	^37^
Lung	PF (idiopathic, bleomycin-induced), ILD, SSc, ARDS, sarcoidosis, PH (monocrotaline- and bleomycin-induced, CDH-associated), environmental exposures (*e.g*., silicosis, asbestosis), airway hypersensitivity/hypersensitivity pneumonitis	Beneficial	^46–48,58,61,64,108^
	PF, PH	Maladaptive	^44,50,109^
Liver	NAFLD, acute hepatitis (ConA-, CCl4-, and LPS-induced), viral hepatitis, cirrhosis (thiocetamide- and CCl4-induced, autoimmune), ALD (LPS- and endotoxin-induced, infectious)	Beneficial	^110–119^
Intestine	IBD, stricturing disease (postoperative/postinfectious)	Beneficial	^13,65,66,68–70,73,74,78,120,121^
	IBD	Maladaptive	^76,77,79^
Pancreas	Acute pancreatitis (cerulean-induced), CP, PDAC, NAFPD	Beneficial	^122–128^
	CP, PDAC	Maladaptive	^10,129–132^
Kidney	TIF/obstructive nephropathy (UUO-mediated), GS, GN, AKI (ischemia-induced), SSc, environmental exposures (*e.g*., tobacco), chronic renal allograft rejection, diabetic microvascular disease	Beneficial	^89,91–96,98,100^

AKI, acute kidney injury; ALD, acute liver disease; ARDS, acute respiratory distress syndrome; CCl4, carbon tetrachloride; CDH, congenital diaphragmatic hernia; ConA, concanavalin A; CP, chronic pancreatitis; GN, glomerulonephritis; GS, glomerulosclerosis; HF, heart failure; IBD, inflammatory bowel disease; IL-10, interleukin-10; ILD, interstitial lung disease; LPS, lipopolysaccharide; MI, myocardial infarction; NAFLD, nonalcoholic fatty liver disease; NAFPD, nonalcoholic fatty pancreas disease; NOS, not otherwise specified; PDAC, pancreatic ductal adenocarcinoma; PF, pulmonary fibrosis; PH, pulmonary hypertension; SSc, systemic sclerosis (scleroderma); TIF, tubulointerstitial fibrosis; UUO, unilateral ureteral obstruction.

**Figure 2.**
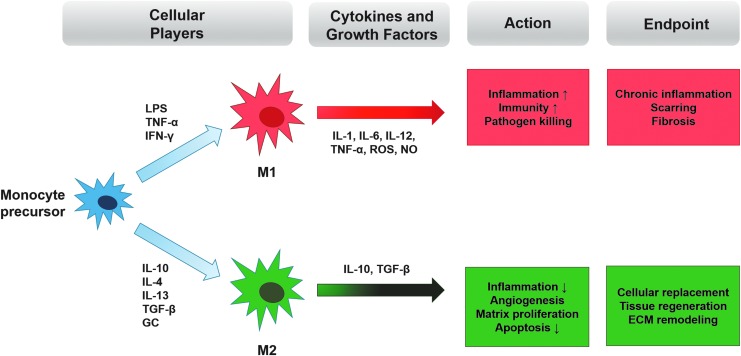
Macrophage polarization. Monocytes are recruited to the site of injury and therein undergo a process of differentiation into M1 (classically-activated) or M2 (alternatively activated) species depending on the microenvironmental cues encountered. The M1 phenotype is induced by immunostimulant molecules such as IFN-γ and bacterial LPS, while the M2 phenotype is induced following exposure to cytokines such as IL-10, IL-13, and TGF-β. M1 macrophage activation and expression of MHC class II antigens propagate further inflammatory signaling and cytokine release, which, despite promoting pathogen killing and immunity, may eventually lead to injury site fibrosis and scarring. M2 macrophages are generally thought to attenuating organ injury by suppressing inflammation and promoting beneficial matrix remodeling and repair, partially via their release of regenerative and anti-inflammatory factors such as IL-10.^99^ GC, glucocorticoids; IFN, interferon; LPS, lipopolysaccharide; MHC, major histocompatibility class; NO, nitric oxide; ROS, reactive oxygen species; TGF, transforming growth factor; TNF, tumor necrosis factor.

IL-10 signals through a tetramer receptor complex (IL-10R) composed of two identical binding subunits IL-10Rα and two homolog signal-transducing IL-10Rβ subunits. Although most hematopoietic cells express this receptor, recent studies have shown that macrophages are the primary targets for IL-10's effects.^9^ Macrophages are frequently characterized as classically (M1) or alternatively (M2) activated species (Fig. 2): M1 cells are induced by antigens such as bacterial cell wall lipopolysaccharide (LPS) or by cytokines such as interferon-gamma (IFN-γ) and tumor necrosis factor alpha (TNF-α) to ultimately produce proinflammatory nitric oxide (NO) and reactive oxygen species (ROS) as host defense mechanisms, including antitumor effects.^10^ In contrast, when M2 cells are activated by IL-4/IL-13 and IL-10 in response to injury, they act to promote wound healing by dampening inflammation and stimulating new ECM formation. In the service of tissue repair, however, M2 cells (and indirectly, IL-10) promote collagen production and, ultimately, fibrosis via transforming growth factor (TGF)-β-mediated fibroblast recruitment and activation. In addition, these and other macrophage subpopulations act to modulate the activity and balance of matrix metalloproteinases (MMPs) and tissue inhibitors of matrix metalloproteinases (TIMPs), thereby controlling the degree of ECM turnover and deposition in the remodeling stage of wound healing. For this reason, Wynn and Barron and others have deemed macrophages as the “master regulators of fibrosis.”^11^ In the literature, IL-10 has been reported to play a critical function in both the classical and alternative macrophage activation processes, although its roles vary and are sometimes opposing.^12^ However, given the high degree of macrophage plasticity and propensity for phenotype switching in response to changing microenvironmental conditions, this contradictory evidence should not be interpreted as inconsistent or conflicting.^10^

More recently, new metabolomic evidence for IL-10's role in macrophage phenotype switching has been discovered.^13^ When activated with LPS, macrophages shift toward a glycolytic metabolic profile, associated with high levels of mitochondrial ROS and aberrant inflammasome activation. The addition of IL-4 or IL-10, however, promotes macrophage commitment to oxidative phosphorylation and thus preserved cellular respiration in the mitochondria. These profound metabolic adaptations to inflammation suggest that they must play a role in macrophage activation and function in different milieus. IL-10's proposed role in controlling macrophage cellular metabolism lies in its indirect inhibition of mTORC1 (mammalian target of rapamycin complex 1) via STAT3 signaling.^13^ As mTOR signaling has been shown to be a central player in the switch from oxidative phosphorylation to glycolysis, its inhibition by IL-10 has thereby been shown to strongly promote autophagy of mitochondria that retain a dysfunctional, inflammatory metabolic program.

Although some studies posit that IL-10 inhibits fibrosis primarily by regulating the inflammatory processes thought to be driving fibroproliferation, the molecular mechanisms behind this stated effect are still incompletely characterized. As previously noted, IL-10 is often considered the dominant anti-inflammatory and antifibrotic player in active inflammation and in wound healing. This unilateral designation, however, fails to take into account the complexity of the process of fibrogenesis itself. In so doing, one can overlook potential therapeutic avenues or even trial harmful ones. For example, IL-10 is clearly an immune-activating cytokine in the treatment of some solid tumors, where it appears to have a stimulating effect on immature T cells by promoting their differentiation into tumor-killing effector T cells.^14^ In addition, long-term IL-10 exposure or IL-10 application to chronic disease processes may actually exacerbate tissue injury and promote fibrotic outcomes, despite being largely beneficial in the setting of acute inflammation and the early stages of wound healing. Furthermore, an overly simplistic view of the cytokine's function fails to recognize the intrinsic harm in immunomodulation, in that bluntly increasing IL-10 levels may limit the appropriate inflammatory response to pathogens and thereby risk infection.

It is clear that IL-10 plays an important regulatory role in health and homeostasis at both the local and systemic level. We will describe recent research into the role of IL-10 in different organ systems, investigating the underlying themes and pathways that lead to fibrosis or regenerative healing. Taken together, this review will reinforce the classification of IL-10 as a complex, multifunctional cytokine that is intimately involved in the fibrotic response to injury, which by further investigation may yield a more accurate picture of its cell- and setting-specific effects, and the therapeutic potential therein.

## Discussion of Mechanisms and Therapies

### Skin fibrosis

**Figure 3.**
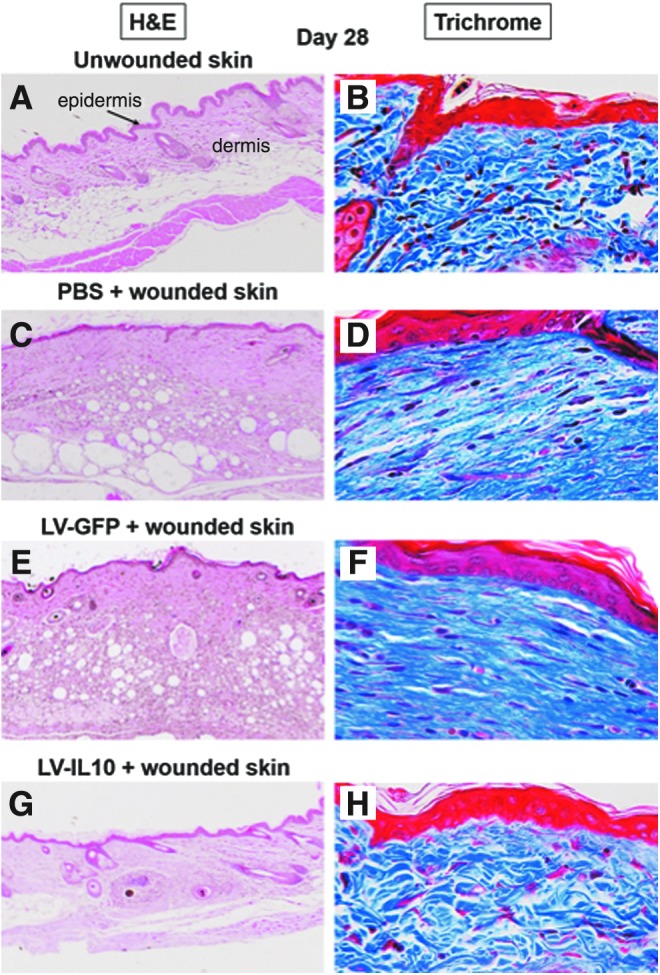
**(A–H)** Representative histology of uninjured murine skin and murine wounds treated with LV IL-10 and controls at 28 days postwounding. Images show H&E staining **(A, C, E, G)** (4 × objective) and Masson's trichrome staining **(B, D, F, H)** (40 × objective) of the wounded/repaired tissue. LV-IL-10 overexpression in murine wounds results in regenerative wound healing **(G)** that is indistinguishable from the surrounding skin, compared to scar formation (flattened epidermis, lack of dermal appendages) in PBS **(C)** and LV-GFP controls **(E)** at 28 days. As shown in **(H)**, Masson's trichrome staining demonstrates that the addition of IL-10 to the wound results not in a scar, but in the restoration of normal ECM architecture **(H)** (basketweave collagen packing in *blue*), compared to thick parallel bundles of collagen in LV-GFP and PBS-treated wounds **(D)**. GFP, green fluorescent protein; H&E, hematoxylin and eosin; LV, lentiviral; PBS, phosphate-buffered saline. Figure reprinted with permission of the author(s) and journal.^22^

Postnatal tissues tend to respond to injury with a well-established series of steps that ultimately result in scar formation. In contrast, fetal wounds sustained into the midgestational period heal regeneratively, or scarlessly, resulting in repaired tissue that is virtually indistinguishable from the uninjured surrounds architecturally, functionally, and mechanically.^15^ Intuitively, then, fetal and postnatal tissues should have critical intrinsic differences that translate into these divergent wound repair outcomes, and significant work has been done to explain and exploit the biologic basis behind this regenerative property. Previous studies have identified aspects of the intrauterine environment, a distinctive fetal fibroblast phenotype, an attenuated inflammatory response to injury with differential growth factor expression, and a uniquely hydrated and anti-inflammatory ECM as essential to fetal regenerative healing.^16,17^ Fetal wounds, in comparison to identical postnatal wounds, are profoundly anti-inflammatory. This is dually observed in the decreased number of immune cells infiltrating the wound and in the decreased expression of proinflammatory cytokines, such as IL-6, IL-8, and TGF-β1 and -β2.^18^ Fetal neutrophils and macrophages continue to respond appropriately to injurious stimuli, which suggests that diminished inflammatory cells at the site of injury may be secondary to lower cytokine expression rather than to immaturity of the prenatal immune system.^19^ Another factor responsible for this attenuated response to injury may be that, at 24 weeks gestation, dermal angiogenesis has not yet completed; thus the initial steps in the postnatal wound healing program, including hemostasis with platelet activation and inflammatory cell homing and invasion, do not completely occur.^20^ Accompanying this decrease in proinflammatory cytokines is a marked increase in the baseline expression of IL-10 in fetal skin and serum, which appears to play a critical role in the regenerative response. This is perhaps exemplified by the observation that fetal IL-10 knockout mouse wounds scar at a gestational age that typically heals scarlessly, with regeneration of dermal appendages.^21^ Studies by our group were the first to demonstrate that IL-10 gene transfer into adult wounds via viral vector resulted in regenerative healing, with an ECM that was visually and biomechanically indistinguishable from unwounded skin (Fig. 3).^21–23^ This suggests that IL-10 not only indirectly modulates fibrosis via its anti-inflammatory properties but may also stimulate fetal-like fibroblast behavior and thus fetal-like ECM production.^21–23^

A second defining feature of the scarless fetal wound environment is the makeup of the ECM, particularly its high concentration of high molecular weight hyaluronic acid (HMW-HA). Once considered a widely distributed but inert structural matrix component, HMW-HA is now considered anti-inflammatory and antifibrotic in its own right, due to its ability to promote TGF-β3 and type III collagen formation in the ECM while also suppressing platelet activation and growth factor release.^24,25^ In addition to its effects on local cytokine and matrix production, HMW-HA has also been shown to inhibit effector T cell activation while enhancing the immunosuppressive effects of regulatory T cells (Tregs), thereby linking the composition of the ECM directly to the adaptive immune response.^26^ Similar to the effect of IL-10 overexpression in postnatal tissues, the addition of HA into postnatal mouse incisional wounds resulted in a wound healing phenotype matching that of a midgestation fetal wound.^17^ As we and others have shown that IL-10 promotes the production of HMW-HA in multiple organs, this may be another avenue by which IL-10 promotes the anti-inflammatory and regenerative capacity of fetal tissues.^22^

Harnessing IL-10 as a therapeutic capable of inducing scarless wound healing, either by promoting its autogenous production *in situ* or by delivering exogenous IL-10, has thus far been a challenge. The cytokine's lability, combined with a lack of enthusiasm for viral vector for use in human disease, has limited the efficacy and applicability of these strategies.^27^ In an attempt to overcome these challenges, our group has developed a novel HA-based, IL-10 impregnated hydrogel that mimics the biochemical milieu characteristic of the fetal ECM.^28^ The glycosaminoglycan components of this topical gel bind IL-10, then allow its slow release into the local surrounds. This hydrogel is inexpensive and easy to use, and we have shown it to successfully recapitulate fetal regenerative healing in postnatal wounds.^28^

### Cardiac fibrosis

The heart is unique in that the endpoint of cellular injury can manifest in two different ways: (1) replacement fibrosis, characterized by cardiac myocyte loss and hyperactivity of cardiac myofibroblasts, and (2) reactive fibrosis, more akin to the multistep fibrogenic process seen in skin and other tissues.^29^ As the heart has little to no capacity to repair or regenerate, normal tissue replacement with scar appears to preserve the heart's functional integrity, although with some significant side effects.^30^ Perhaps the most damaging is the replacement of normal electrical conduction pathways by fibrotic tissue, which is significantly arrhythmogenic.^29,31^ Even small increases in the degree of wall and septal scarring are associated with a significant increase in the risk of cardiac events.^32^

The role of IL-10 in limiting cardiac injury and fibrosis is rather indirect. Specifically, IL-10 and its downstream signaling pathways, notably STAT3, are critical in recruiting and retaining bone marrow-derived endothelial progenitor cells to the site of heart injury, whose stem-like properties then influence repair and regeneration.^33,34^ Noncardiomyocytes appear to provide the majority of IL-10 expression in the heart, but the effects of IL-10 knockout are experienced at all levels of cardiac tissue, reinforcing the importance of even indirect IL-10 signaling: worse histologic and clinical outcomes occur after both local (myocardial ischemia-reperfusion models) and systemic (LPS injection) insults in these animal models.^35^

The Kishore group has done extensive work delineating the role of inflammation in myocardial injury states. In a model of acute myocardial infarction, IL-10 administration significantly suppressed proinflammatory cytokine production, MMP-9 activity, and inflammatory cell infiltration of the myocardium, with a resultant decrease in cardiac fibrosis.^7^ This resulted in improved left ventricular function and diminished pathological remodeling, including smaller infarct size and less wall thinning. The same group demonstrated a cardioprotective effect of IL-10 in both a surgical model of cardiac hypertrophy and heart failure and in IL-10 knockout mice with isoproterenol-induced pressure overload.^36^ Recombinant IL-10 administration improved ventricular function, decreased hypertrophic remodeling, attenuated cardiac fibrosis, and reduced mortality. These effects were shown to be dependent on STAT3 signaling and the inhibition of proinflammatory gene expression.^36^

In contrast, the paradoxical role of IL-10 in fibrosis is exemplified by a recent study by Hulsmans *et al.* on the pathogenesis of heart failure with preserved ejection fraction,^37^ wherein human patients and mouse models of the disease demonstrated excess cardiac macrophage numbers and IL-10 production. Certain subsets of cardiac resident macrophages (MHCII^high^) were shown to release IL-10 in response to systemic inflammation from left ventricular diastolic dysfunction, leading to an autocrine loop that promoted a new, fibrogenic macrophage phenotype, one that secreted osteopontin and TGF-β. These mediators were shown to activate cardiac fibroblasts to secrete profibrotic cytokines and attract immune cells that ultimately promoted collagen deposition, exacerbating cardiac stiffness and diastolic dysfunction. Deleting macrophage-derived IL-10 resulted in both decreased fibrosis and improved clinical indicators of heart function. They conclude by suggesting that systemic neutralization of myeloid-specific IL-10, either directly or by modulating macrophage phenotypic changes, may be a promising therapy in this specific subset of patients via its indirect effect on cardiac remodeling.

Recently, IL-10 has also been examined for its therapeutic role in atherosclerosis. Atherosclerotic lesion development is a stepwise chronic inflammatory process involving dysregulated ECM remodeling. It begins with endothelial dysfunction and disruption, enabling circulating lipoproteins to invade the intima and recruiting T lymphocytes to the site of injury. Monocytes/macrophages also translocate into the arterial wall at the site of the fatty streak, ultimately transforming into lipid-laden foam cells, a process that initiates atheromatous plaque formation. Smooth muscle cells, induced by platelet-derived growth factor (PDGF), TGF-β, and other growth factors released by foam cells and activated endothelia, migrate from the media to the intimal plaque, where they produce matrix components that encapsulate the lesion (the “fibrous cap”).^38^ At the same time, however, cytokines released in response to this inflammatory process stimulate foam cell macrophages to secrete MMPs; these enzymes degrade collagen and elastin and thereby compromise the integrity of the newly synthesized fibrous cap. Accumulation and apoptosis of these foam cells in combination with unchecked matrix remodeling leads to progressive plaque growth and weakening, ultimately resulting in an unstable and thrombogenic necrotic core and eventual plaque rupture. To potentially intervene in this process, Pinderski *et al.* sought to more closely examine the pathogenesis of a previously observed therapeutic role for IL-10 in inhibiting the development and progression of atherosclerotic lesions.^39–42^ They first developed a transgenic mouse model wherein IL-10 overexpression is localized only to activated T lymphocytes, then successfully engrafted transgenic or wild-type bone marrow into low-density lipoprotein receptor-deficient mice—a phenotype that induces constitutive hyperlipidemia—fed an atherogenic diet. The mice experiencing T cell-mediated IL-10 overexpression had notably suppressed initiation and progression of atherosclerotic lesions, with a decrease in lesion size, complexity, and inflammatory oxidation products in the vessel wall. These results were seen in the setting of normal plasma IL-10 levels, localizing the effect to the level of the transgenic lymphocytes. In addition, both foam cell apoptosis and IFN-γ expression by activated macrophages were decreased in the transgenic mouse, reinforcing the influence of monocyte-mediated effects on inflammation and fibrosis and IL-10's critical role in regulating those effects. Because this work demonstrates a beneficial role for IL-10 on multiple immune cell types in all stages of atherogenesis, the therapeutic implications are quite far-reaching.

### Pulmonary fibrosis

In healthy lungs, alveolar macrophages are the primary source of IL-10, secreted constitutively under homeostatic conditions and upregulated in disease states, as seen upon LPS or TNF stimulation. T cells are a secondary, but critical, source for the cytokine in inflammatory conditions. A beneficial role for IL-10 has been well described in acute inflammatory conditions such as asthma and acute respiratory distress syndrome, but its role in diseases marked by pathological fibrosis is less clear.^43–48^ These include both restrictive diseases of the pulmonary interstitium, perhaps best exemplified by idiopathic pulmonary fibrosis (IPF), and pulmonary vascular pathologies, as seen in the fibrotic vascular remodeling that characterizes pulmonary hypertension.

IPF is a life-threatening and progressive restrictive lung disease with limited therapeutic options. Most patients die within 5 years of diagnosis secondary to complications of hypoxic respiratory failure, which results from the progressive matrix deposition in the pulmonary interstitium. The accepted story for the pathogenesis of IPF has undergone significant revision in recent years. Rather than just the end product of nonspecific repetitive alveolar damage, IPF is now viewed as an example of aberrant wound healing after transient injury, wherein dysregulated crosstalk between the alveolar epithelial cells and the ECM after tissue injury comes to a fibrotic conclusion.^49^ The cells responsible for this fibrotic matrix appear to include resident lung fibroblasts and myofibroblasts, the source(s) of which may include primary resident cells, those produced secondarily via epithelial–mesenchymal transition (EMT), and potentially circulating fibrocytes.^50^

Both a pro- and anti-inflammatory cytokine, TGF-β appears to play a major role in this dysregulated crosstalk.^51^ Like IL-10, it is also produced by alveolar macrophages, and has been shown to induce fibroblast proliferation, differentiation into myofibroblasts, and subsequent collagen production seen in the fibroblastic foci that mark IPF lungs.^52^ TGF-β is known to be a critical inducer of EMT and the related process endothelial-to-mesenchymal transition, in which epithelial or endothelial cells acquire a profibrotic mesenchymal phenotype under inflammatory pressure.^53^ Activated lung fibroblasts can then induce alveolar cell apoptosis, worsening the phenotype and perpetuating the cycle of aberrant activation.^29^ These known effects are already being pharmacologically exploited in human disease: pirfenidone, an inhibitor of TGF-β production and activity, has been approved by the Food and Drug Administration for patients with IPF for its ability to slow disease progression and improve survival.^3,54^ Overall, the literature supports a symbiotic or interdependent relationship between IL-10 and TGF-β, including the ability to induce and modulate each other's production in T cells.^55–57^ Viewing IL-10 through the lens of its effect on TGF-β-induced signaling pathways may help better define the potential mechanism of action behind IL-10's pleomorphic role in IPF and other fibrotic processes.

**Figure 4.**
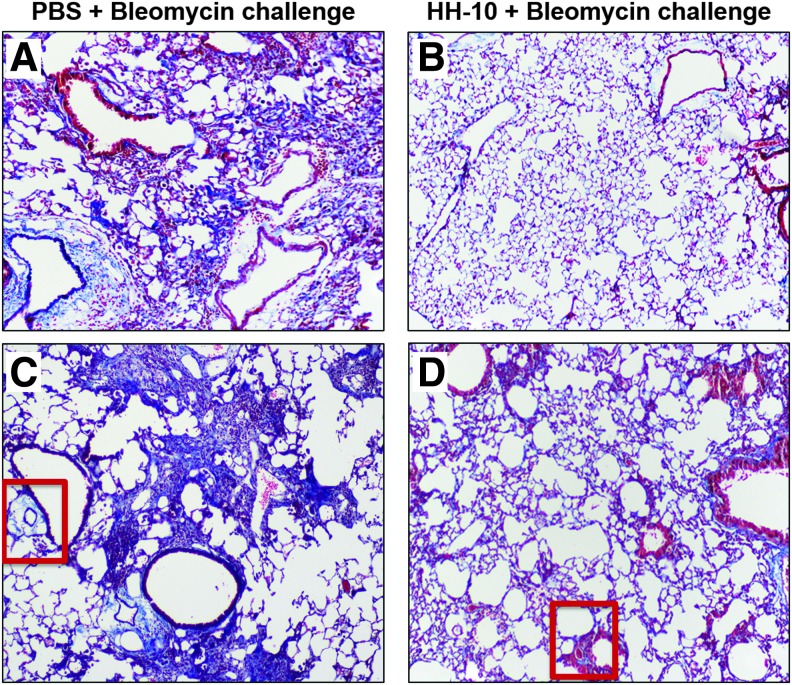
**(A–D)** Representative histology of bleomycin-challenged murine lungs, with and without intranasal HH-10 hydrogel treatment. Intranasal administration of bleomycin induces pulmonary fibrosis in mice and is an established experimental model of human IPF. Compared to PBS/control-treated animals **(A, C)**, 7 days of intranasal HH-10 (200 ng/mL IL-10 in a hyaluronan-based hydrogel) treatment **(B, D)** decreases the size and severity of fibrotic lesions in the lungs of bleomycin-challenged mice. Note also that the extent of perivascular fibrosis, as demonstrated by the extent of *blue* staining (*red boxes*) was also reduced by treatment with HH-10. Images show trichrome staining (*top row*: 4 × objective; *bottom row*: 40 × objective) of lung tissue sections of bleomycin-challenged treatment cohorts. IPF, Idiopathic pulmonary fibrosis. Sample images courtesy of S. Balaji and V. de Jesus Perez, with results as described in Shamskhou *et al*.^59^

On the basis of this relationship and on IL-10's known anti-inflammatory properties, Nakagome *et al.* used intratracheal bleomycin to model IPF, then administered intravenous IL-10 plasmid 2 weeks later, mimicking the treatment of established or chronic disease. IL-10 was shown to suppress the development of pulmonary fibrosis in a TGF-β1-dependent manner, wherein TGF-β1 production by alveolar macrophages was suppressed by IL-10 both *in vivo* and *ex vivo.*^58^ A similar model, but one that avoids the confounders inherent in using systemic IL-10, is used by Vinicio de Jesus Perez's group, wherein murine bleomycin-induced pulmonary fibrosis is both prevented and reversed after treatment with an intranasal IL-10/hyaluronan-based hydrogel (Fig. 4).^59^ The limitation of these models, however, is that pulmonary fibrosis resolves when bleomycin administration stops, unlike in human disease.

The spatiotemporal dependence of IL-10's mechanism of action and effects is well-demonstrated in a series of studies wherein short-term and long-term lung-specific tetracycline-inducible IL-10 overexpression in transgenic mice was compared. Short-term overexpression demonstrated decreased pulmonary inflammation after LPS insult, while long-term IL-10 overexpression actually increased lung fibrosis by increasing the recruitment of and infiltration by T cells, B cells, M2 macrophages, and collagen-producing fibrocytes.^50,60,61^ This recruitment was associated with high CCL2 (C-C motif chemokine ligand 2)/CCR2 (C-C motif chemokine receptor 2) expression, a cytokine axis associated with M2 macrophage differentiation and with the development of pulmonary fibrosis in other mouse models: for example, CCR2 null mice exposed to bleomycin do not develop IPF.^62^

Fibrotic pulmonary vascular remodeling, as opposed to interstitial fibrosis, leads to pulmonary arterial hypertension (PAH): a disparate group of diseases characterized by elevated pulmonary artery pressures and increased resistance to blood flow, ultimately leading to right heart failure and death. Current front-line vasodilatory therapies are inadequate, as most do not target the fibrotic ECM that develops around the pulmonary resistance vessels and limits compensatory vessel dilation. High baseline levels of IL-10 are seen in the serum of patients with PAH, which could be interpreted as a protective anti-inflammatory measure occurring in response to an ongoing injury process.^43^ Interestingly, patients with severe PAH being treated with intravenous prostacyclin agonists actually had higher circulating IL-10 levels compared with untreated counterparts.^63^ Whether these observations suggest that PAH target therapies amplify an immunoprotective response or, conversely, that IL-10 is a marker of disease severity, is unclear. Much of the experimental data in the literature, however, suggest a beneficial role for IL-10 in pulmonary hypertension. In a rat model of monocrotaline-induced PAH, Ito *et al.* administered IL-10 via intravenous adenoviral vector, which resulted in significantly improved survival rates and a reduction in mean pulmonary artery pressures.^64^ In addition, we and our collaborators have described a beneficial role of IL-10 delivery in treating pulmonary hypertension. In a mouse model of congenital diaphragmatic hernia that is notable for developing clinical and histologic pulmonary hypertension at 4 weeks of life, we show that treatment with an IL-10/HA hydrogel reduced the perivascular inflammatory cell burden and lowered pulmonary vascular resistance (unpublished data).

### Intestinal fibrosis

Several studies have identified multiple cellular sources of IL-10 in the gut, namely activated macrophages, anti-inflammatory type 2 helper T cells, and human intestinal epithelial cells.^65,66^ Intestinal IL-10 production is thought, as in other organs, to promote tissue integrity and regulate inflammation after injury by inhibiting the effector functions of activated immune cells.^67^

Inflammatory bowel disease (IBD) comprises Crohn's disease and ulcerative colitis (UC), both chronic diseases of the small and large intestine with complex pathogeneses and polymorphic and multiorgan presentations. Classically, Crohn's disease is more strongly associated with full thickness injury of the small bowel leading to fibrosis and stricturing, while UC is associated with colonic disease and refractory GI bleeding. As with any autoimmune disease, in IBD, there is a loss of self-tolerance, either to commensal bacteria or intestinal neoantigens, and there appears to be a significant homeostatic role for IL-10 in maintaining intestinal tissue integrity and suppressing inflammation.^68^ In addition, because both peripheral blood monocytes and resident intestinal monocytes in IBD patients produce higher levels of proinflammatory cytokines than normal patients, and because bone marrow transplantation has been shown to effect IBD remission, intestinal tissue damage can be said to be due to both systemic and local causes.^69^ In further support of the centrality of IL-10 in IBD, IL-10 knockout mice exposed to nonsterile laboratory conditions invariably develop chronic enterocolitis, with symptoms that improve with exogenous IL-10.^70^ In humans, defective IL-10 proteins and/or IL-10 signaling are associated with both UC and Crohn's: low levels of IL-10 in ileal tissue of patients with Crohn's disease is associated with disease recurrence, and IL-10 polymorphisms have been shown to confer a higher risk of IBD.^13,71–74^ Interestingly, systemic IL-10 levels do not appear to be associated with the presence or severity of IBD.^75^ Unfortunately, IL-10 immunotherapy in treating IBD, particularly Crohn's, has had mixed results in multiple clinical trials. Endoscopic and clinical improvements in mild Crohn's after treatment with subcutaneous IL-10 depots were shown to be strikingly dose dependent: while moderate dose levels of systemic IL-10 had favorable clinical results, higher doses actually resulted in elevated plasma levels of the inflammatory markers IFN-γ and neopterin in treated patients.^4,76–78^ A 2010 Cochrane review of multiple trials of recombinant IL-10 revealed little benefit to the treatment and recurrence of Crohn's while noting the prevalence of significant adverse effects from the therapy leading to significant trial subject dropout.^79^

Although treating IBD with IL-10 directly has not been wholly successful, insights into the mechanism of action behind the anti-inflammatory and immunomodulatory roles of IL-10 more globally could inform the design and implementation of novel future therapies for this challenging disease. Ip *et al.* has recently shown that macrophages from the intestinal lamina propria of IL-10-null mice and from IL-10 receptor-deficient IBD patients with colitis accumulated mitochondria with higher ROS levels, aberrantly secreted IL-1β, and demonstrated increased mTORC1 and inflammasome activation, all of which strongly contributed to intestinal inflammation.^13^ Mice with colitis treated with antioxidants or rapamycin, an mTOR inhibitor, showed suppressed IL-1β secretion. They conclude that targeting the mTORC1 pathway in macrophages in an effort to eliminate those with dysfunctional mitochondria, whether by modulation of that pathway with IL-10 treatment or otherwise, may present a significant therapeutic target in IBD and related intestinal inflammatory disorders.

Some of the aforementioned challenges of delivering IL-10 in an effective manner are negated by using Tregs—a subgroup of T cells that largely serve to quell inflammation—that have been genetically engineered to express the cytokine of interest as a delivery vehicle.^80^ Using an immune cell as an intermediary logically increases the tissue specificity of the deliverable, in that IL-10 would be directly transported to the site of inflammation, thereby increasing target tissue bioavailability while avoiding undue systemic side effects. In addition, the gene transduction step would proceed *ex vivo*, negating concerns about viral and retroviral vector use in human subjects.^81^ Furthermore, Tregs in themselves have been shown to play a beneficial role in maintaining immune control of the intestinal mucosa, with IL-10 playing an essential role in their activation and function.^82–84^ The gut-specific regulatory function of IL-10 produced by these cells is seen in mice with Treg-specific ablation of a conditional IL-10 allele: these mice spontaneously develop colitis, but not systemic autoimmunity.^85^

To this end, van Montfrans *et al.* transduced CD4^+^ T lymphocytes with retroviral vectors expressing IL-10 and green fluorescent protein (GFP) upon activation. The majority of these cells were shown to have gut-homing potential, based on mucosal adhesion molecule expression, and were also shown to decrease major histocompatibility class (MHC) II expression as well as dendritic cell production of IL-12 *in vitro*.^86^ When the transduced T cells were introduced into the CD45RB^high^ mouse model of colitis, intestinal inflammation was effectively prevented, even when given 2 weeks postcolitis induction. Demonstrating that these T cells homed to the site of intestinal inflammation and had a therapeutic effect *in vivo*, IL-10 was detected in the colon and its lymph node drainage basins in the weeks after transduction, and similarly, colonic and nodal TNF-α production was decreased in the colon of the transduced mice.^87^ In contrast to these striking findings, the same transduced T lymphocytes had no effect on acute trinitrobenzene sulfonic acid (TNBS)-induced murine colitis. Consequently, the use of modified T cells and/or Tregs as an effective and tissue-specific strategy for IL-10 delivery in IBD has yet to progress past the experimental stage.

### Renal fibrosis

Both the classic elements—inflammatory cells and the cytokines they release—as well as the kidney's unique architecture and intrarenal signaling pathways, such as the renin-angiotensin-aldosterone system (RAAS), play a critical role in the reaction to and resolution of kidney injury. Injury occurring in the tubules, interstitium, or glomeruli/capillaries may elicit different initial reactions (*e.g*., glomerulosclerosis or tubulointerstitial fibrosis), but ultimately the end point of all injury is disproportionate ECM deposition and function-limiting fibrosis.

**Figure 5.**
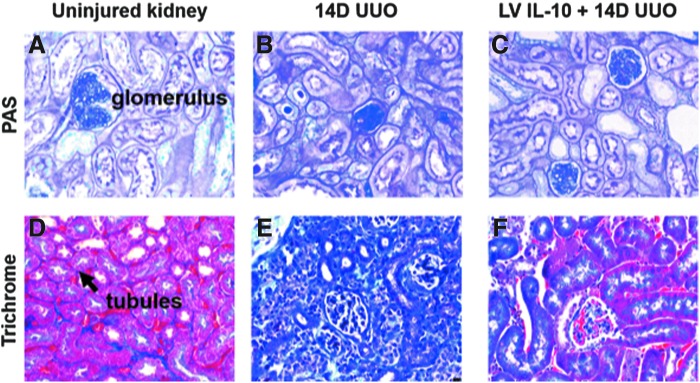
**(A–F)** Representative histology of control/normal and 14 days post-UUO murine kidney cortices, with and without LV IL-10 treatment. Images show PAS staining **(A–C)** (40 × objective) and trichrome staining **(D–F)** of control, untreated UUO, and IL-10-treated UUO kidney cortices. 14 days of ureteral obstruction results in significant kidney injury **(B, E)** marked by glomerular involution, tubular dropout, and increased interstitial spaces filled with collagen (*blue*/*purple*). With the addition of LV IL-10 **(C, F)** injected into the renal parenchymal 3 days before injury, the inflammatory damage and fibrosis levied by UUO is markedly reduced. PAS, periodic acid-Schiff; UUO, unilateral ureteral obstruction. Data and figures courtesy of X. Wang; article under review for publication.

Almost three decades ago, the beneficial negative-feedback properties(s) of IL-10 were identified in multiple heterogeneous kidney pathologies and was shown to be highly expressed by diseased glomeruli.^88–90^ Mu *et al.* reported that IL-10 reduced fibrosis in a rat model of chronic kidney disease (CKD), attributing it to the cytokine's immunosuppressive function.^91^ Further reinforcing the critical role of IL-10 in renal homeostasis, Jin *et al.* demonstrated severe tubular injury with increased inflammation and collagen deposition in an IL-10-deficient mouse, as well as upregulated profibrotic markers such as alpha-smooth muscle actin (α-SMA).^92^ The authors suggested a role for TGF-β signaling pathways, including EMT, in the pathogenesis of renal fibrosis in this model. In addition, IL-10 also appears to have an effect on profibrotic signaling pathways that are unique to the kidney, specifically that mesenchymal stromal cell-released IL-10 inhibited RAAS signaling and thereby reduced tubular scarring after unilateral ureteral obstruction (UUO).^93^ As in the heart, the molecular mechanism behind this effect appeared to be a decrease in inflammatory cytokine mRNA due to HuR transcription inhibition. Similarly, our laboratory has shown that exogenous IL-10 administration post-UUO attenuates tubulointerstitial fibrosis and is additionally associated with upregulated HMW-HA, similar to the effects seen with IL-10 in the skin. A representative image of IL-10's ability to attenuate UUO-induced renal fibrosis is seen in Fig. 5 (data from article under review; sample images courtesy of X. Wang).

While there have been promising results in animal models, translating those results into the treatment of human disease has been more of a challenge, due to IL-10's short half-life and rapid renal elimination. As in the skin, various groups have administrated IL-10 to kidney disease models via viral gene transfer and in hydrogels.^94–97^ As in colitis, some of the challenges of efficient IL-10 administration have been avoided by using *ex vivo* modified immune cells, and in so doing also serve as a possible insight into IL-10's complex and interconnected role in the pathophysiology of kidney fibrosis. Indeed, novel therapies for renal fibrosis are not limited to direct application of IL-10 itself: Romero *et al.* reported that l-citrulline, an alpha-amino acid, significantly increased IL-10 levels and resulted in remarkably reduced tubulointerstitial fibrosis in a type I diabetic mouse model.^98^ Some cell-based therapies have also had remarkable results. The literature has previously reported on innovative renoprotective interventions exploiting macrophage polarization as a way to condition the ECM at the site of injury and effect an antifibrotic outcome.^99^ Some of the most notable work in this field has been done by Harris and colleagues, wherein splenic macrophages were modified *ex vivo* with IL-10/TGF-β to induce or polarize to an anti-inflammatory, antifibrotic M2 phenotype.^100^ These M2s were then transfused into a mouse with Adriamycin nephropathy, a condition considered analogous to human focal segmental glomerulosclerosis. The activated macrophages were shown to protect against renal inflammation and subsequent structurofunctional damage in this mouse model of CKD. In addition, the M2 macrophages induced Foxp3+ Treg activity and proliferation both *in vitro* and *in vivo* via costimulatory molecule B7-H4 signaling, while also inhibiting CD4^+^ and CD8^+^ effector T cell proliferation. These macrophages did not to switch phenotypes (*i.e*., M2 to M1) *in vivo*, and in fact were shown to deactivate existing endogenous renal proinflammatory M1 macrophages. Nonetheless, no matter the basis of delivery, these lines of exploration could benefit the millions of patients in the United States and worldwide that suffer from the effects of CKD.^70^

## Summary

In summary, IL–10 has complex, pleomorphic, and sometimes opposing effects depending on organ system, disease state, and cell type from which it is derived, as well as multiple venues for external regulation of its expression. Because of the highly plastic nature of fibrosis itself, the sometimes contradictory effects of IL-10 in this process should not come as a surprise, but the more intriguing aspect of IL-10 signaling is the conserved mechanisms by which it appears to act in multiple organs, as demonstrated by the studies we have highlighted (Table 1). Understanding these conserved, but complex, signaling mechanisms involving IL-10 could uncover new potential therapeutic targets for the design of improved therapies for fibrosis in multiple organs.

**Take Home Messages**

- Fibrosis is a common final pathway of many diseases, accounting for nearly 50% of mortality worldwide.
- Past its known anti-inflammatory properties, IL-10 signaling is a conserved mechanism regulating fibrotic processes in multiple organs.
- IL-10 signaling is pleiotropic, and its effects are dependent on its cellular source, the organ system involved, and the local milieu.
- Novel therapies have been developed to deliver recombinant IL-10 or to use cell-based strategies to intervene in IL-10 signaling pathways to prevent or minimize fibrosis.
- Further research on IL-10 signaling and its context-dependent effects could lead to new therapeutic targets for antifibrotic treatments.

## About the Authors

**Emily H. Steen, MD,** is a general surgery resident at Baylor College of Medicine completing her research fellowship in the Laboratory for Regenerative Tissue Repair (LRTR). **Xinyi Wang, PhD,** is a postdoctoral fellow in the LRTR. **Swathi Balaji, PhD,** is an Assistant Professor of Surgery in the Division of Pediatric Surgery at Baylor College of Medicine/Texas Children's Hospital. **Manish Butte, MD, PhD,** is an Associate Professor in the Department of Pediatrics and Division Chief of Immunology, Allergy, and Rheumatology at the David Geffen School of Medicine at UCLA. **Paul L. Bollyky, MD, DPhil,** is an Assistant Professor in the Division of Infectious Diseases of the Department of Microbiology and Immunology at Stanford University School of Medicine. **Sundeep G. Keswani, MD, FACS, FAAP,** is the Surgical Director of Basic Science Research, Professor of Surgery, Pediatrics, and Obstetrics and Gynecology at Baylor College of Medicine/Texas Children's Hospital, and the Principal Investigator of the LRTR.

## Abbreviations and Acronyms

CKD: chronic kidney disease
ECM: extracellular matrix
EMT: epithelial–mesenchymal transition
HA: hyaluronic acid (hyaluronan)
HMW: high molecular weight
HuR: human antigen R
IBD: inflammatory bowel disease
IFN: interferon
IL: interleukin
IPF: idiopathic pulmonary fibrosis
LPS: lipopolysaccharide
LRTR: Laboratory for Regenerative Tissue Repair
M1: classically-activated macrophage
M2: alternatively activated macrophage
mRNA: messenger RNA
PAH: pulmonary arterial hypertension
RAAS: renin-angiotensin-aldosterone system
ROS: reactive oxygen species
TGF: transforming growth factor
TNF: tumor necrosis factor
UC: ulcerative colitis
UUO: unilateral ureteral obstruction
